# Delay enhancement cardiac MR imaging for assesment of myocardial involvement in patients with systemic lupus erythematosus and clinical suspect of myocarditis

**DOI:** 10.1186/1532-429X-11-S1-P271

**Published:** 2009-01-28

**Authors:** Sandra Graciela Rosales Uvera, Leticia Castellanos Cainas, Jaime Galindo Uribe, Jorge Vazquez La Madrid, Jorge Oseguera Moguel, Martha Morelos Guzman

**Affiliations:** National Institute of the Medical Science and Nutrition Salvador Zubiran, Mexico D.F., Mexico

**Keywords:** Systemic Lupus Erythematosus, Cardiac Magnetic Resonance, Myocarditis, Cardiac Damage, Left Ventricle Mass

## Introduction

**Figure 1:**
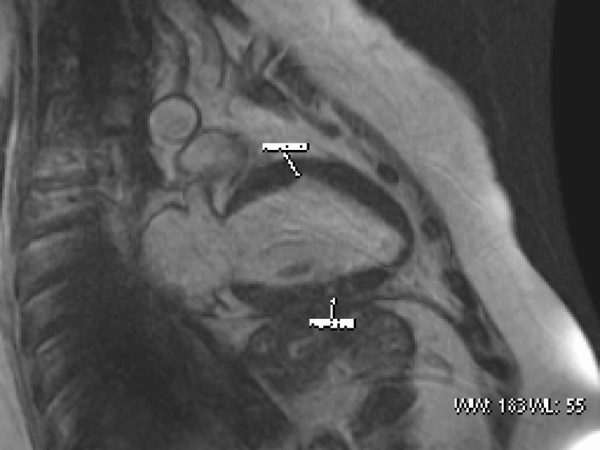
**37 year old female with 3 year evolution of SLE and myocarditis, with precordial pain and dispnea, left ventricle is dilated, low ejection fraction and fibrosis with patchy pattern, other sings of damage were mitral and aortic regurgitation, biatrial enlargement and pericardial effusion**.

The systemic lupus erythematosus (SLE) is a multiorgan inflammatory autoimmune disease mainly affecting women and is associated with high cardiovascular motality; myocarditis is a rare complication; however little knowledge about cardiac damage by myocarditis in patients with SLE. The cardiac magnetic resonance (CMR) is a useful tool for diagnosis of myocarditis and evaluate progression or regression of cardiac damage caused by SLE. See Figure 1.

## Purpose

The purpose of this preliminary study is to evaluate the structural and functional alterations in patients with SLE and clinical suspect of myocarditis.

## Methods

9 SLE consecutive patients (mean age 40.5 years), 1 man and 8 woman. Diagnosis followed the criteria of American Rheumatism Association; assessment of SLE activity was based on the European Consensus Lupus Activity Mesurement (ECLAM) Index. Underwent CMR on a GE 1.5 tesla magnet for assessment of cardiac function, cine images used a balanced steady state free precession (FIESTA) technique with retrospective ECG triggering (TE = 1.6 TR = 3.6, flip angle 45 =, slice thickness = 8 mm) short and long axis images were performed, matrix 224 × 224. Ten minutes after administration of 0.2 mmol/kg of Gd-DTPA a *k* space segmented 2D inversion-recovery gradient echo secuences (in-plane resolution 1.3 × 1.3 mm2, slice thickeness 8 mm) with complete coverage of the left ventricle (LV) myocardium in short axis slices and additional long axis view. Images were analyzed based on 17 segment model to evaluate segmental wall motions abnormalities.

## Results

Mean LV end-diastolic volume 147 ml and mean LV end-systolic volume 75 ml with LV dilated in 2 patients, mean LV mass 109 gr, mean left ventricular ejection fraction was 51.8%, 77% have segmental wall motions abnormalities, 55% left and right atrium enlargement. Aortic valve alterations in 55%, mitral valve involvement 100%, pericardial effusion 66%. Delay enhancement imaging revealed intramyocardial hyperenhancing lesions in the LV myocardium in 4 patients (44%) without relationship with coronary artery territory.

## Conclusion

Preliminary data suggest delayed enhancement CMR is avaible to detect cardiac damage caused by SLE myocarditis, besides CMR can identify valvular damage, atrial enlargement and pericardial alterations as early evolution of the disease.

